# Vulnerability to high risk sexual behaviour (HRSB) following exposure to war trauma as seen in post-conflict communities in eastern uganda: a qualitative study

**DOI:** 10.1186/1752-1505-5-22

**Published:** 2011-10-19

**Authors:** Wilson Winstons Muhwezi, Eugene Kinyanda, Margaret Mungherera, Patrick Onyango, Emmanuel Ngabirano, Julius Muron, Johnson Kagugube, Rehema Kajungu

**Affiliations:** 1 Makerere University College of Health Sciences, School of Medicine, Department of Psychiatry, P. O. Box 7072, Kampala, Uganda; 2 Medical Research Council/Uganda Virus Research Institute (MRC/UVRI), P. O. Box 49, Entebbe, Uganda; 3 Transcultural Psychosocial Organization Uganda (TPO-Uganda), Plot 3271 Kansanga off Ggaba Road, P.O. Box 21646 Kampala, Uganda; 4 Uganda Bureau of Statistics (UBOS), P.O. Box 7186, Kampala, Uganda; 5 Butabika National Referral Mental Hospital, Plot 2, Block 237-238, Butabika Road, Kampala, Uganda

## Abstract

**Background:**

Much of the literature on the relationship between conflict-related trauma and high risk sexual behaviour (HRSB) often focuses on refugees and not mass in-country displaced people due to armed conflicts. There is paucity of research about contexts underlying HRSB and HIV/AIDS in conflict and post-conflict communities in Uganda. Understanding factors that underpin vulnerability to HRSB in post-conflict communities is vital in designing HIV/AIDS prevention interventions. We explored the socio-cultural factors, social interactions, socio-cultural practices, social norms and social network structures that underlie war trauma and vulnerability to HRSB in a post-conflict population.

**Methods:**

We did a cross-sectional qualitative study of 3 sub-counties in *Katakwi *district and 1 in *Amuria *in Uganda between March and May 2009. We collected data using 8 FGDs, 32 key informant interviews and 16 in-depth interviews. We tape-recorded and transcribed the data. We followed thematic analysis principles to manage, analyse and interpret the data. We constantly identified and compared themes and sub-themes in the dataset as we read the transcripts. We used illuminating verbatim quotations to illustrate major findings.

**Results:**

The commonly identified HRSB behaviours include; transactional sex, sexual predation, multiple partners, early marriages and forced marriages. Breakdown of the social structure due to conflict had resulted in economic destruction and a perceived soaring of vulnerable people whose propensity to HRSB is high. Dishonour of sexual sanctity through transactional sex and practices like incest mirrored the consequence of exposure to conflict. HRSB was associated with concentration of people in camps where idleness and unemployment were the norm. Reports of girls and women who had been victims of rape and defilement by men with guns were common. Many people were known to have started to display persistent worries, hopelessness, and suicidal ideas and to abuse alcohol.

**Conclusions:**

The study demonstrated that conflicts disrupt the socio-cultural set up of communities and destroy sources of people's livelihood. Post-conflict socio-economic reconstruction needs to encompass programmes that restructure people's morals and values through counselling. HIV/AIDS prevention programming in post-conflict communities should deal with socio-cultural disruptions that emerged during conflicts. Some of the disruptions if not dealt with, could become normalized yet they are predisposing factors to HRSB. Socio-economic vulnerability as a consequence of conflict seemed to be associated with HRSB through alterations in sexual morality. To pursue safer sexual health choices, people in post-conflict communities need life skills.

## Introduction

Globally, armed conflicts result in unprecedented waves of population displacement, both within and across borders. Armed conflicts are associated with direct consequences like deaths, diseases, stress, migration and environmental destruction; indirect socio-economic disruption in the form of institutional, infrastructural and human capital destruction; and opportunity costs like famine due to disruption of agriculture as well as poverty due to disruption of commerce and education [1]. In Uganda, large parts of the north and east of the country display signs of impoverishment, possibly because of devastation associated with recent armed conflict [2]. Out of an estimated total population of 30.7 million people, the 2009/10 Uganda national household survey estimated that by region, the poor in the north were 46.2%, 24.3% in the east, 21.8% in the west and 10.7% in the central [3].

Compared to other regions in Uganda, more people in the north and east of the country were displaced from their homes and exposed to high risk sexual behaviours (HRSB). Generally, displacement of people is associated with food insecurity, sexual exploitation especially by men who wield some form of power (economic, physical and social), gender-based sexual violence, idleness and drunkenness and compromised resilience. Due to long stays in camps, avoidance of risky sexual behaviour is more likely to be compromised. Other likely consequences of people's displacement include inadequacy of income and other basic needs, severe deprivation leading to commercial sex work, chaotic circumstances in which access to condoms and other preventive options may be scarce, and lack of health infrastructure and education. These factors are known to expose people, especially women and children to HRSB [4-7]. HIV/AIDS prevalence rates in conflict-affected areas of Uganda were reported to be higher than the national average of 6.4% [7]. For instance, unlike in other rural regions, the prevalence rate of HIV/AIDS in North Central Uganda (Acholi, Teso, Lango) was found to be 8.2%, which is comparable to urbanized Kampala and the Central region of 8.5% [5,7,8]. This could partly be explained by the over 20 years of war between the Government of Uganda and the rebels of the Lord's Resistance Army (LRA).

The UNAIDS (Joint United Nations Programme on HIV/AIDS) Inter-Agency Task Team on Gender and HIV/AIDS reports that 75% of the more than 35 million people made refugees or displaced by conflict globally are women and children [9]. This exposure to conflict and associated trauma is likely to be associated with HRSB [10,11]. High risk sexual behaviour (HRSB) refers to any lifestyle or an activity that places a person or people at an increased risk of suffering or getting infected with HIV/AIDS, a sexually transmitted disease and/or an unwanted pregnancy. In this article, HRSB was taken to include engaging in one, some or all the following behaviours; extra marital sex, multiple sexual partners, cross-generational sex, transactional sex, a high turnover of sexual partners, sex with uniformed personnel, and unprotected sex with persons whose HIV sero-status is suspected to be positive [12,13].

In Sub-Saharan Africa, HRSB and violent conflicts are known to interact in shaping population health in dramatic ways. HRSB may be created by conflict [14,15]. HRSB may also affect the epidemiology of HIV/AIDS [16,17]. This is well explained by the theoretical perspectives of ecological systems theory which explains human behaviour in terms of forces at individual, social, political, cultural, and other levels and not merely the level of individual psychology [18,19]. The theory posits that understanding the dynamics underlying any human behaviour requires an examination of the social systems within which risky behaviour occurs and underscores the fact that behaviour is influenced not only by the social context but also the social support systems, level of conflict and social interactions. In Northern Uganda, a recent study established that traditional social institutions that influence behaviour and regulate sex were rendered dysfunctional by conflict and displacement, thereby paving way for HRSB [5,7].

Pervasive conflict and war often catalyze the disintegration of communities and families as well as the disruption of social norms governing people's sexual behaviour [20]. Men who lose their status in their communities or families due to armed conflict are more likely to resort to alcohol abuse and to engage in HRSB. Most women also become vulnerable given their increased dependence on men for physical or economic security. Since many displaced persons are forced to leave their homes, women may be forced to trade sex with armed men or other people supposed to protect them in exchange for food, water, shelter, protection and other basic commodities. Such "survival sex" might involve sex with men infected with sexually transmitted infections (STIs), including HIV. Women are also likely to suffer at the hands of boys and young men who become child soldiers and are forced to become violent and abusive as part of their training [9].

Globally, much of the literature about the relationship between conflict-related trauma and HRSB is on refugees and not mass in-country displacement of people by ensuing armed conflicts which is the focus of research for this article. Secondly, though a lot of research in the area of HIV/AIDS in non-war affected communities in Uganda abounds, there is little known about conflict and post-conflict communities. Therefore, it was worth every effort to examine the interrelationship that may exist between prolonged conflict and HRSB among the people of *Teso *sub-region in Eastern Uganda.

Understanding factors underpinning vulnerability to HRSB in the various high-risk populations is an important cog in the design of interventions for HIV/AIDS prevention. Using a qualitative approach, this study investigated the dynamics underlying vulnerability to HRSB following exposure to war trauma as seen in Katakwi district, Eastern Uganda. Specifically, this article reports results of a study that examined; (i) the interrelationship between vulnerability, exposure to war trauma and HRSB and (ii) the socio-cultural dynamics that underlay risky sexual behaviours in a post-conflict population.

## Methods

### Study Site and Context

The study was conducted in *Teso *sub-region districts of Katakwi and Amuria among people who were the target of a community psychosocial intervention project implemented by Transcultural Psychosocial Organization (TPO-Uganda) under the auspices of Uganda AIDS Commission-Civil Society Fund. The main aim of the project was to reduce the risk of HIV infection among war affected vulnerable groups. This project was encouraging the uptake of HIV prevention measures. The TPO-Uganda project had the following activities;

1. Screening for exposure to war trauma, psychological and gynaecological effects of war trauma, membership to various vulnerability groupings, and HRSB

2. Providing rehabilitation through community interventions like encouraging membership to support groups, attending of community counselling programs, psycho-education programs and general health education

3. Providing out reach medical services at health centres for people with more severe forms of mental health and gynaecological problems (including STI) as a consequence of war trauma, and

4. Providing a package of HIV prevention services like; HIV/AIDS voluntary counselling and testing (VCT), health education about HIV/AIDS, encouraging health seeking behaviour for sexually transmitted disease (STDs), facilitating provision of services for STD treatment and life skills training for in-school and out-of-school youths.

TPO-Uganda is a Non-Governmental Organization (NGO) that commenced operations in Uganda in 1994 with the aim of providing psychosocial support and mental health care to communities, families and individuals in conflict and post-conflict settings. TPO-Uganda has projects in the West Nile, Northern and in Eastern regions of Uganda.

The study participants were from 4 sub-counties, 3 in Katakwi district and 1 in Amuria district. By 2010, the estimated population of Katakwi was 153,600 people and 315,900 people in Amuria [21]. Media reports in Uganda suggested that by the end of 2005, the prevalence of HIV/AIDS in Katakwi had increased from 9% to 21% [22]. Secondly, anecdotal information from the district put the prevalence rate of HIV/AIDS and Sexual Reproductive Health (SRH) problems at 17% [23]. However, the total prevalence of HIV/AIDS in northeast Uganda where Katakwi and Amuria are two of the 7 districts was put at 3.5% by a national survey [8]. The main ethnic groups in the area are the *Iteso *and *Kumam *and the main language is *Ateso*. Subsistence agriculture and pastoral animal husbandry are the two main economic activities. The agricultural products are millet, sorghum, groundnuts, sim-sim, cow peas, maize, soya beans, sweet potatoes, and vegetables. The main cash crop is cotton. Though having tarmac roads, the two districts have mostly seasonal, dusty and pot-holed roads and footpaths traversed mostly by motorcycles and bicycles.

Contextually, the conflict in the study area had existed in varying intensities and intermittency from as far back as the 1950's. The main players were: *Karamonjog *cattle rustlers; Uganda People's Army (UPA) who staged a rebellion against the government from 1985 to 1992; Alice Lakwena's spiritual/political rebellion of 1987-1988; Lord's Resistance Army (LRA) incursions in the *Teso *sub-region in 2002, 2004 and 2006; and government armed forces who participated in each of the conflicts by trying to re-establish normalcy and liberate civilians [24-26]. By January 2010, about 200,000 people out of about 2 million who had been displaced in northern Uganda and the Teso sub-region were still living in camps [27]. In the research for this article, the opinion leaders, community members and guides from TPO-Uganda helped in selection of potential participants.

### Design of the Study

We used a cross-sectional qualitative approach to examine the dynamics that underlay HRSB in trauma-affected populations and people's perceptions, beliefs and explanatory models. The approach is known to be good in understanding social processes and concepts from the perspectives of study participants informed by their lived experiences [28]. This qualitative study was part of the bigger study conducted to assess vulnerability to HRSB following exposure to war trauma as seen in Eastern Uganda.

### Study Participants and Procedures

Data were concurrently collected between March and May 2009 from non-probabilistic purposive samples of 32 key informants, 16 in-depth interviews and 8 FGDs who were equally spread in the study area. Selection of study participants was done in such a way that it represented variation in the phenomenon of interest. The various sources of data and number of study participants are summarized in Table 1. All study participants had to be aged 15 years and above which is closer to 15.7 years, the median age of sexual debut for females in East Central Uganda where Katakwi and Amuria are found) [29]. They also had to be residents in the study area. To attain theoretical sensitivity, heterogeneity of participants was ensured through adherence to diversity in terms of age and gender differences of selected study participants.

**Table 1:** Sources of qualitative data and study participants

Method	Age category	Sex of study participants	Districts			
			Katakwi District (A)			Amuria District (B)
			Sub-county A	Sub-county B	Sub-county C	Sub- county D
Focus Group Discussions (FGDs)	15-24 years	Men	1			
		Women	1			
	25-34 years	Men		1		
		Women		1		
	35 Years and more	Men			1	
		Women			1	
	In-school youths	Men				1
	Out of school youths	Women				1
	Total FGDs		2	2	2	2

†Key Informants (KIs)	Elders' representatives		1	1	1	1
	Cultural leaders' representatives		1	1	1	1
	IDP camps leaders		1	1	1	1
	Religions leaders		1	1	1	1
	School head teachers		1	1	1	1
	District political leaders		1	1	1	1
	Local Council leaders		1	1	1	1
	Civil Society Organization representatives		1	1	1	1
	Total KIs		8	8	8	8

#Case Studies		Men	2	2	2	2
		Women	2	2	2	2
			4	4	4	4

Notes

**#**Case studies involved people who were perceived by the community opinion leaders and members to have been exposed to too much trauma

† The key informants were 50% men and 50% women

Whereas the possibility of compromising data quality was high, this was anticipated and forestalled during the planning process for fieldwork. The data collection tool was pre-tested to ensure its appropriateness. The tool was originally formulated in English but translated into *Itesot*, and blind back-translated into English to ensure conceptual consistency and accuracy. Research assistants who spoke both languages translated the guide from English to *Itesot*, and the other four assistants did the back-translation.

Before collecting any data, participants were told about the study purpose. Data was collected and analyzed to a point where no more new information to enrich theme identification was forthcoming [30]. Theme identification in the data started during literature review and continued as long as patterns that captured interesting issues were emerging [31,32]. We started to notice and look for patterns of meaning and issues of interest like the interface between civilians and the armed forces, parental loss as a precursor to HRSB, food scarcity, changing socio-cultural configuration due to conflict, poverty and transactional sex, cultural breakdown, fatalism, and learned helplessness, and trauma-induced consequences as data collection progressed. The process of data collection stopped when we began to notice repetition of information--almost verbatim--from different study participants.

#### Focus Group Discussions (FGDs)

Using open-ended questions, FGDs were conducted with men alone and women alone in the age ranges of 15-24, 25-34 and ≥ 35 years. Additional 2 groups were of in-school and out of school youths. The FGDs were conducted at venues convenient to study participants, in *Ateso*, the local language dialect. FGDs ensured collection of data on attitudes, perceptions, beliefs and practices regarding vulnerability to HRSB following exposure to war trauma in the study communities. The FGDs consisted of 7 to 12 participants and were useful in eliciting rich data associated with peer interaction and debates.

#### Key Informant interviews (KIs)

Key informants were expert sources of information, who given their personal skills, or position within a society, were able to provide more information and a deeper insight into what was going on around them. They were the "natural observers" and were interested in the behaviour of those around them. They could observe the development of their culture and make inferences [33]. We used this method to collect data from elders, cultural leaders, leadership of internally displaced peoples' (IDP) camps and other opinion makers like religions leaders, district political and civil leadership and civil society organizations (CSO) workers in the study area. The purpose of these interviews was to obtain in-depth information on the study phenomenon.

#### In-depth case study interviews

Sixteen case studies half of whom were coping with war-related trauma rather poorly were purposively selected and engaged in in-depth interviews. The case-studies were drawn from all the four study sub-counties and had both male and female representation.

Data collection was based on appropriate interview guides based on a review of an earlier version of the McGill Illness Narrative Interview (MINI) [34], field experience, and research objectives. The first author developed the data collection guides, each of which had a section consisting of some demographic profile, vulnerability narrative, context of trauma and sexual behaviour, explanatory models of sexual behaviour in a post-conflict setting, and impact of conflict-related trauma on sexual behaviour as domains of inquiry shown in Table 2.

**Table 2:** Questions asked during data collection

Domain of inquiry	Probable questions to ask
Socio-demographic profile	District & Sub-county
	Village/Camp
	Site
	Number of participants
	Moderator
	Note taker
	Date
	Starting time
	Ending time
	Transcriber
	Gender (number of men or number women for FGD)
	Age (age range for FGD)

Vulnerability narrative	As a group, when do you think you experienced life difficulties associated with conflict/war for the first time? What happened then? And then?]
	Which life difficulties associated with conflict/war have you experienced in your lifetime?
	We would like to know more about your experiences of life difficulties associated with conflict/war, Can you describe what happened when you faced life difficulties?
	Looking at your history, can you tell us how your well-being/lifestyles used to be before you experienced life difficulties associated with conflict/war?
	What have been the changes in your well-being/lifestyles after you experienced the life difficulties associated with conflict/war?

Context of trauma and sexual behaviour	Are there any other people in your social environment *[family, social environment like friendship networks] *who experienced/are experiencing life difficulties associated with conflict/war like you? In what ways do you consider your life difficulties similar or different to theirs?
	Have you ever heard [on radio, from other people] or read [newspapers] about any other person or people who had the same life difficulties' associated with conflict/war as you? [If yes] In what ways is such a person/are such people's life difficulties' similar or different to yours?
	Please, tell us about the self-protecting things/resources which used to be available to protect against life difficulties when your people would be face traumatizing events? [Probe traditional ways of managing life difficulties]
	Which resources *[social and political networks and institutional] *are available to protect against life difficulties when people face traumatizing events?
	Looking at your history, can you tell us how sexual decency/integrity in your society used to be preserved before life associated with conflict/war befell your home areas?
	Looking at your current society how has life difficulties associated with conflict/war affected sexual decency/integrity?

Explanatory models of sexual behaviour in a post-conflict setting	Do you have a local terms or expression that describes your life difficulties associated with conflict/war? [What is that local term or expression?]
	According to you, what do you think are primary causes of your life difficulties? *[Take special interest to note all that is mentioned]*
	Was/is there something happening in your family, at work or in your social life that could explain your life difficulties associated with conflict/war? *[If answer is yes, as next question]*
	In your society, how do you deal with [manage, cope] life difficulties associated with conflict/war? What assistance or help is given to someone facing a life difficulty?
	Are your life difficulties somehow linked or related to specific events that occurred in your lives?

Impact of conflict-related trauma on sexual behaviour	How have your life difficulties associated with conflict/war changed your life/lives?
	How has your life difficulty associated with conflict/war changed the way you feel or think about yourselves?
	How has your life difficulty associated with conflict/war changed the way you look at life in general? *[Probe for impact on sexual behaviour]*?
	What has been happening to your social and sexual lives ever since you faced the life difficulties associated with conflict/war?
	How have your lifestyle difficulties associated with conflict/war changed the way others look at you?
	How have life difficulties affected people's motivations, beliefs, values, practices and opinions about sexuality in this community?
	For those who faced lifestyle difficulties associated with conflict/war and coped well, what can explain the resilience/hardiness/toughness?
	How has you family or friends helped you helped you through this difficult period associated with conflict/war in your life?
	How have your spiritual lives, faiths or religious practices helped you go through these difficult periods in your life?
	Is there anything else you would like to add?

The research team discussed and reached a consensus on suitability of the questions and pre-tested them on similar participants that were excluded from the final study. Research assistants were trained on all aspects of qualitative research notably: how to collect data, how to probe and paraphrase and ethical obligations in research. The purpose of training was to ensure that they gained ability to get exhaustive and in-depth data about study participants' full stories regarding the sexual behaviour.

Each data collection session was tape-recorded after seeking permission of study participants. Data was thereafter transcribed verbatim by a bilingual speaker following acceptable guidelines [31]. All research assistants also took detailed field notes. Data collection was overseen by a supervisor who coordinated four teams of research assistants, each consisting of a moderator and a note taker. Each team was assigned a sub-county. Each member of the research team was required to keenly observe the context of the study population and note down salient features. This was necessary to back-up and contextualize the data that was collected.

### Ethical considerations

We obtained ethical and administrative clearances from the Uganda National Council for Science and Technology Committee on the Study of Human Subjects, the administrators of the study districts and the local community leaders. Study participants gave verbal informed consent to be interviewed and we assured and accorded them privacy, anonymity, and confidentiality. We told them of their liberty to withhold information they were uncomfortable to give. We referred those who asked questions requiring therapeutic answers to appropriate professionals for help.

### Data analysis

Analysis and collection of data progressed simultaneously. Emerging themes and impressions guided data collection. Words, content and context of what was said by participants was analyzed to 'get a mental picture' of the interrelationship between war-related trauma and HRSB. Data were analyzed and interpreted manually. The *Ateso *audiotapes of all the data collection processes were transcribed following standard guidelines [35,36] into English, scrutinized, and categorized by a bilingual speaker. Transcripts were reviewed and checked against original audio recordings by a language expert to ensure translation accuracy. The transcribed data were compiled into a text document. The first author closely read each transcript several times to get familiar with the depth and breadth of the data content, inscribe notes on margins of the data book, identify key words, search for more meanings and patterns, and write detailed notes on emerging themes [31,32].

Coding was approached, in part, with questions that inspired the study and also with an expectation of coming across novel information. Through systematically working through the whole data set, coding progressed from what was available at data collection to assortment into identified patterns [31,32]. After identifying several codes, we matched them with comparable 'chunks' of data extracts. Examples of codes identified included; **'**orphan-hood resulting from deaths associated with cattle rustling activities and with HIV/AIDS', 'interaction between civilians and the military', 'a socio-cultural environment associated with HRSB', 'a high population density in IDP camps', 'transactional sex and HRSB', 'changes in sexual practices', 'cultural breakdown', 'alcohol abuse', 'early marriages', 'forced marriages', 'learned helplessness', 'gender-based violence' and many others. After generating a list of codes and collating them with data extracts, they were sorted into potential sub-themes and themes. Observed differences and similarities within the data aided in assigning different data segments to different tentative themes.

Each extract of transcribed data was subjected to thematic analysis [37]. Through constant comparison, emergent themes, sub-themes, and data extracts coded were identified [31,32,38]. Some tentative themes could be accommodated in others while others deserved to be broken down. Bearing in mind the objectives of the study, theory and literature; data content, study context and underlying clusters of concepts, and relationships between codes, themes, and different levels of themes were noted [32]. Thereafter, more review and refinement was conducted to ensure coherent patterns [31]. The final themes were; (i) breakdown of the social structure in the study population, (ii) consequences of exposure to conflict on resilience, (iii) gender-based abuse in a post-conflict setting and (iv) the relationship between exposure to prolonged war trauma, well-being and HRB. These themes appeared to be linked to HRSB and are discussed in the results section. We used illuminating verbatim quotations from participants to illustrate major findings.

## Results

In keeping with the ecological systems theory that looks at the influence of several environmental systems on an individual [18], this study set out to explore the relationship between conflict-related trauma and sexual behaviour of individuals using a qualitative research design. Some of the study participants had left IDP camps and resettled in their original villages. Others were still living in IDP camps. The outcome of analysis and interpretation of findings are the social phenomena summarised in the final themes.

### Breakdown of the social structure in the study population

Data from case studies, FGDs or KIs demonstrated high levels of socio-economic and cultural adversity that seemed to be related to vulnerability to HRSB. Reports from study participants indicated that people in the study area had faced adversities ranging from loss of livestock to cattle rustlers, food insecurity, forced soldiering, early marriages, loss of livelihoods and overcrowded living arrangements in IDP camps characterized by squalid living conditions, dysfunctional families and break down of social ties.

### Discrediting of sexual sanctity in society

Even after coming out of displacement that came about due to ensuing conflicts and wars, most people in the study population were still mourning the loss of their property which had always been a basis of their livelihood. Apparently, this had forced some of them to adopt HRSBs to acquire basic necessities of life. With the maltreatment of men during the conflicts who are traditional breadwinners, many widows and vulnerable girls were reported to have ended up with no viable alternatives of supporting their families. Engaging in transactional sex was reported to have become a choice for many. Many of them took to engaging in HRSB to get money to buy food or to get physical food from those privileged to control or own it. Even after the 'guns had fallen silent' many widows and vulnerable girls were known to have continued with the HRSB adopted during the time of active conflicts. Findings from an FGD of 12 men aged 24-35 years and a 45 year old female who was a case study showed that impoverishment associated with the conflict had increased HRSB;

. . . with onset of the wars, things here changed . . . poverty increased, insecurity became an issue and, we could not live freely like it was before. Soldiers started to use money to lure young girls and women into sex . . . HIV/AIDS became common here when soldiers were deployed to guard our camp . . . many started sleeping with our daughters and wives . . . in our communities, whenever a new person would arrive in a camp, women would be easily enticed by him into sex . . .

. . . living in IDP camps affected us . . . our cultural values and norms lost their sting . . . children stopped respecting their elders . . . they started sexual acts early. In the crowded camps, children would see their parents in sexual acts because the huts were too small for all family members . . . if a woman failed to get something from a husband and someone else was there and willing to do it in exchange for sex, she would go for it. This became common . . . most men lost their wives and daughters to soldiers . . .

In the context of history and nature of the ensuing conflict, study participants described the gradual disintegration that their lives took over a period of time. The types of lifestyles which preceded the conflict were nostalgically described as having been blissful and characterized by respect for elders and sexual sanctity. During the course of the wars and conflict, factors that demeaned sexual sanctity like poverty, concentration of many people in IDP camps, civilian-military interaction, and transactional sex were thought to have become unrelenting as illustrated the words of a 45 year old representative of a community based organisation (CBO) reproduced below;

Ever since problems befell our community, people's lives have been affected negatively . . . spread of diseases like HIV/AIDS became rampant due to crowding in IDP camps . . . the spread of HIV in this place started with deployment of soldiers here. Soldiers took people's wives since they had more money . . . these women came back to their husbands with HIV.

Similar sentiments were affirmed by participants in a 9-member FGD of men aged 24-35 years. For some men, displacement into IDP camps had also meant loss of power and ability to provide for their families. That is perhaps why some made references to wives or daughters that were taken over by other men who had money and influence.

It looks plausible that the civil conflict resulted in socio-economic and cultural impoverishment with many implications on sexual behaviour. The post-conflict socio-economic and cultural setting still had signs of living in conflict notably; food insecurity, poor and overcrowded housing, learned helplessness, and apathy. A high level of impoverishment is known to drive vulnerable groups in society, like women into adopting HRSB like transactional sex in order to secure survival for either themselves or their children. The destruction of means of livelihoods associated with conflict had indeed pushed some women into HRSB in order to support themselves and their families. According to an FGD of men aged 24-35 who were still in an IDP camp, this had dire consequences on sexual behaviour;

. . . our lives here have become are characterised by difficulties . . . we cannot cultivate easily as we used to . . . we lack what to eat . . . as a result of overcrowding in the camp, evils like sexual immorality have spread . . . we have very many cases of defilement, extra marital sex, early marriages and forced marriages, many of which were unheard of in the past . . . because they want to look smart and to have beautiful things, our girls and even women are enticed by those with money into having sex. . .

### Perceptions about sexual morality and decency

The exposure to conflict for a long time was believed to have had implications on people's sexual integrity as well as the timing of marriage. Although this exposure was experienced by both women and men, women and adolescent girls appeared to have faced the heaviest brunt of trauma associated with conflict. Men and women believed that some of the socially nauseating sexual practices like incest had crept into the current society due to alterations in the socio-cultural context. This sentiment is well expressed by a 42-year old man in sub-county A, the FGD of men aged 24-35 years, and the FGD of women aged 24-35 years in that order;

So many things like incest, defilement, forced marriages and early marriage . . . are rampant in our society. These things used to be rare in our society . . . displacement by wars lead our people to learn strange behaviours . . . parents have started forcing their daughters to marry early because they want money . . . Young people got to know about sex very early in life . . . young girls and boys learnt to go for old men and women simply because a young person would take pride that a man or woman can provide for his or her needs

. . . our neighbouring community of cattle rustlers led to many deaths and forced us into IDP camps. These neighbours are partly responsible for today's forced marriages and other sexual immoralities in our area . . . If they had not forced us into camps, sexual immorality, infidelity, defilement and rape wouldn't have become common here . . . nowadays, people force their sons to get married early before the raiders come to steal the cattle. Secondly, parents force their daughters to marry such that they can get replacement of the stolen cattle.

. . . sex is being misused, it is an embarrassment to the society . . . young men nowadays go into sexual relationship with their mothers' age-mates, girls have started to have sex with old men. Sex has been commercialized . . . It has become worse with the worsening economic turmoil where everything needs money. Unwanted pregnancies have become common and so are abortions

### Transactional sex in the context of vulnerability

At the peak of the conflict, the level of interaction between civilians in the study area and newcomers like military personnel and aid workers was reported to have been high. This happened in a population with many orphans and other vulnerable groups of people who had been created by adversities associated with cattle raids, abductions and murders. Other than adults who engage in transactional sex due to economic challenges, orphans especially of the female sex in the emerging individual-centred study population are known to end up in HRSB like prostitution and early marriages because of societal neglect. The views of a 49-year camp leader who was an elder and the FGD of younger men aged 15 to 24 years respectively illustrate the assertion above;

. . . we have people living with HIV and different kinds of orphans . . . for some, one of the parents is dead while for others, both are dead . . . this is a problem . . . most of the girls who are orphans go for early marriages so as to be able to support their siblings . . .

. . . life in IDP camps has really doomed us . . . because of behaviour adopted while in camps, sex has ceased to be respected . . . Sexual encounters now happen anywhere i.e. in pit latrines, bathrooms and even in open places giving children the opportunity to watch and thus get motivated to try doing it too. . . married women cannot afford basic needs for their children . . .in IDP camps, they learnt that they can survive if they get fairly rich men to have sex with in exchange for the basic provisions of life, thus increasing the risk of acquiring or spreading HIV . . . young girls learnt to be in love with elderly men for benefits like school fees, food at home and scholastic materials.

### Consequences of exposure to conflict on resilience

Due to life adversity associated with conflict, some of the initially resilient people ultimately succumbed to engaging in HRSB. The words of a 40-year old man from sub-county B reproduced below illustrate how most men were forced into life trajectories known to be associated with engaging in HRSB.

. . . in October 1987, the cattle rustlers killed my father . . . . I dropped out of school. . . . In 1990, we were hit by famine and our mother abandoned us . . . I struggled to bring up my siblings since I was the first born. I married early because I needed someone to help me take care of my siblings . . . In 1996, I joined the army but the pay was not good and I used to be transferred quite a lot. I stopped soldiering when my two brothers were in secondary school. In 2005, I tested HIV positive and . . . I started septrin prophylaxis. I have since had other confirmatory HIV and Tuberculosis (TB) tests . . .

A number of social ills like; apathy to work, thinking about committing suicide, and land conflicts were common in the study population and looked to have been associated with exposure to traumatic conflicts. The effect of different people coming to live together in IDP camps where there was little or no work seemed to have cultivated learned helplessness and laziness. Redundancy undermined resilience and predisposed people to substance and alcohol abuse and indulging in HRSB as illustrated by the words of a 65 year old community based organization (CBO) representative;

HIV might have not spread widely as it is nowadays . . . if it was not because of displacement. People would have been in their traditional homes . . . the laziness common in our people is linked to what happened to them . . . there was too much idleness and taking alcohol . . . insecurity made it hard to go back to villages to dig . . .

Concentration of many people in IDP camps was associated with too much idleness. The only pastime for most of the men that were idle in IDP camps was alcohol consumption. Alcohol consumption is known to compromise decision-making which goes hand-in-hand with engaging in HRSB like casual sex. This issue is well captured in the words of a village chairperson in sub-county D;

The lifestyle in IDP camps encouraged over drinking alcohol and engaging in HRSB. People would take advantage of making others drunk to have sex with them . . .

### Gender-based abuse in a post-conflict setting

Women and girls were more likely to be victims of sexual abuse associated with the war and conflict. Reports of girls and women that had been victims of rape and defilement at the hands of rebel fighters and cattle rustlers were common as illustrated by a quotation from a FGD of younger men aged 15 to 24 years from sub-county A;

. . . seeing one's own mother or sister being raped, murdered and starved was too painful . . . The LRA brutally murdered, looted and abducted children and adults, some of whom have never returned . . .

Similar sentiments were expressed by the FGD of younger women from the same age range and sub-county;

". . . the violent approach of the conflict involved raping women and girls and this left many of the victims feeling psychological pain and disgust with their lives . . ."

Similarly, the FGD of women aged 35 years and above from sub-county D shared the same perspective as illustrated below;

. . . innocent people were killed and this left many of them puzzled and confused. Some women and girls were raped and this affected them psychologically and in general health . . . some got infected with diseases they did not have before . . .

Vulnerability to HRSB during war and the belligerent conflict appeared to have been closely knit with role expectations of men, women and children in a home. Women were reported to have persevered in their primary care-giving roles for a decent survival during war even in situations of diminished life opportunities. While pursuing these roles, some women and girls were reported to have ended up engaging in HRSB so as to acquire resources to support either their families or themselves. Some women and girls in the study population were reported to have engaged in HRSB in exchange for food, money, clothes, cosmetics, and other survival requirements. Since some women had no alternatives of meeting their basic needs and those of their families during the intense conflicts, they engaged in HRSB as illustrated by the words of a 50 year old CBO representative from sub-county B, a 49 year old camp leader from sub-county A and a 72 year old elder from sub-county C in that order;

. . . Sex totally lost meaning . . . women started to have sex for material gain and to support their families. . . poverty drove people crazy at the peak of war here! . . . you would find some one giving in to have sex because she wanted to earn a living . . . IDP camp life made the situation worse . . .

. . . all the deviance in sexual behaviour mainly resulted from difficulties that people faced at the peak of war . . . there was food shortage and women started to exchange sex for food, gifts and other valuables. Girls started to be prematurely given out for marriage because of poverty . . . orphaned teenage girls started to just give in to marriage as the only way to have a house. . .

Due to having just come out of a war situation, people generally feel dejected and hopeless, others feel they are the unluckiest in the world . . . some see no reason to live on . . . many women now exchange sex for food and other valuable items. Some have no fear of HIV/AIDS since unlike starvation; it will not kill them immediately . . . these are indications of despair.

Other than sex abuse, cases of gender-based abuse and harassment in the post-conflict study area were not an exception. One thing known to spark gender-based violence is suspicion of marital infidelity. In the study, some men recounted how soldiers, aid workers and businessmen had lured their wives or daughters into sex escapades. Circumstances related to the unrelenting conflict therefore seem to have compromised marriages. As a consequence, domestic violence and misunderstandings seem to have been likely consequences as illustrated by a quotation from an interview with a 21-year old woman from sub-county D reproduced below;

. . . late in 2008, my husband tested HIV positive but for me, I tested negative . . . my husband puts me to task to explain how it happens that I am negative. He claims that I brought the disease . . . He abuses me, beats me and makes my life miserable . . . prolonged war and life in the IDP camp had lead us into many fights leading to separation . . . I married another man . . . my husband also married another woman . . . with return of peace, I went back to our home village and I went back to my original husband . . .

### Relationship between exposure to prolonged trauma, well being and HRSB

Key informants were asked to reflect on the likely impact of exposure to war trauma on people's general well-being. The main finding was that after prolonged exposure to trauma, most people stated to display persistent emotional turmoil and to abuse alcohol. Out of despair and hopelessness, they resorted to HRSB for their comfort as encapsulated in verbal excerpts from a 48-year old man from sub-county A and a 42-year old woman from sub-county C below;

The level of alcohol use for most people more than doubled and this happened after many people from different backgrounds settled in large IDP camps . . . among the youths, a lot of cross-generational sex started to happen . . . young girls started to have sex with old men. . . sexual immorality used to be low before the war . . incidence of rape has increased and so has divorce.

. . . people are settling back from IDP camps into their homes but . . . they still have emotional and psychological problems as a result of their displacement . . . many had become reckless on issues to do with sex because of hopelessness in IDP camps . . . many of them lost respect themselves . . . they used sexual encounters as a way of dealing with emotional pain and feeling joyful even when they would be suffering hence putting their lives at risk of HIV and STIs

## Discussion

We set out to investigate the interrelationship between exposure to war trauma, and HRSB in a post-conflict population. The findings elucidate the susceptibility and vulnerability of people in post-conflict societies to HRSB. Similar to what has been observed elsewhere, exposure to war trauma appears to have destroyed the local economy in the study area [39]. Other than the perceived reduction in the stocks of cattle which lead to impoverishment in the study area, social ills like orphan-hood, widowhood and child-headed households had increased. Orphan-hood of especially the girl child is known to be a predisposing factor for HRSB and consequently HIV/AIDS [5]. Other adversities which people had faced ranged from food shortage, forced soldiering, forced marriages, early marriages, loss of employment, squalid and overcrowded IDP camps, and break down of social ties.

In many stable societies, sexual behaviour is ideally regulated by cultural norms and values that condemn premarital sex, premarital pregnancy and uphold virginity. Some widows and adolescent girls in the study communities that had started to emerge out of a long conflict were reported to be engaging in HRSB in order to access resources for survival. Poverty arising from exposure to conflict in the context of a high concentration of better facilitated newcomers like armed personnel and aid workers in the conflict area seemed to have intensified transactional sex which is one form of HRSB. Civilian-military interaction is a known catalyst of HRSB [4,40].

To a vulnerable and traumatized population, transacting sex for security is cited as one of the factors that explain higher rates of HRSB and HIV infection in IDP camps compared to other parts of society [6,41]. In the study, having sexual relationships with newcomers like armed personnel could have conferred short term gains like security, money, social status/prestige and contentment to the vulnerable people. Already, past research from war-torn northern Uganda had observed that women and girls were more vulnerable and at risk of engaging in HRSB than their male counterparts because of having limited access and control over the much needed resources, especially food [5,7].

Lifestyle in the era preceding conflict in the study area was nostalgically described as having been blissful and characterized by observance and respect for elders and sexual sanctity. One of the consequences of conflict is displacement and concentration of people in IDP camps. In sub-Saharan Africa and elsewhere, IDP camps even when there is no conflict or war are known to be characterized by over-crowding, extreme poverty and unemployment due to 'idleness' all of which are conditions linked to HRSB [42]. In the study area, sexual health-compromising dynamics like civilian-military interaction and transactional sex had become all-encompassing. It appears that there were real changes in people's sexual behaviour marked by increases in casual sexual encounters, infidelity, sexual predation, incest, multiple sexual partners, early marriages, forced marriages, and many others. This was attributable to conflict-related stress, changing norms about acceptable sexual behaviour and peer pressure. Second, with the waning of conflict, resettlement of people who had been dispersed could have started to bring together people with different sexual histories which might increase HRSB as it has happened elsewhere [13].

The socio-economic and cultural impoverishment of people in the study area had implications on sexual behaviour. It is known that poverty often forces some people like women into transactional sex for economic survival [7]. Besides, poorer women are known to have a high likelihood of being raped and sexually tortured [7,43-46]. The high levels of social disadvantage associated with conflict-related losses like; food shortage and alterations in sexual values while in IDP camps seem to have had dire consequences on sexual behaviour. This is in agreement with past research which noted that in conflict settings; poverty, insecurity and gender inequalities usually increase and play a role in perpetuating HRSB [47-49]. These factors compromise individuals' abilities to make personal choices and to negotiate safer sex or abstain.

In the post-conflict society studied, many social ills like apathy to work, land conflicts, and gender-based abuse were reported to be common and perceived to be linked to exposure to war trauma. Concentration of many people in IDP camps with little or no work seemed to have cultivated in that population fatalistic tendencies and learned helplessness, which are known to be predisposing factors to alcohol abuse and risky sexual behaviour [50]. Alcohol is known to inhibit rationality and decision making and it may diminish consciousness towards; negotiation for safer sex, abstinence or having protected sex by using condoms [7,51].

Given the insidious conflict in the study area, some people had been forced to make career choices known to be associated with HRSB. The marauding rebels of armed groups, particularly Karimajong cattle raiders, the LRA and the UPA perpetrated war trauma by instilling fear. The marauding rebels traumatized communities through different ways notable among which were; abductions, cutting off body parts, forcing people to kill and killing in humiliating ways as reported elsewhere [43,44,52]. These rebels were undisciplined militias with a high propensity to commit acts of sexual violence like rape with impunity. As a consequence, many young people were recruited into armed forces either to defend their society against rebels or to seek for alternative means of survival means. A soldiering career in itself is a known risk factor for HRSB [4,6].

Cases of gender-based abuse in the study area were reported. Gender-related abuse is likely to be experienced by women more than men and it is also known to impact negatively on the health and sexual behaviour of mostly women and girls [53,54]. A review of mental health problems among women with a history of intimate partner violence in the United States reported that victims had a 3 to 5 times greater likelihood of depression, suicidality, posttraumatic stress disorder (PTSD), and substance abuse than non-victims [55]. It has also been documented that exposure to trauma and PTSD are associated with negative behaviours such as substance use or abuse and failure to engage in preventive strategies like safe sex [41]. In a situation of war, the possibility of sexual abuse for women and girls is known to be high, especially when they go to the bushes to look for food, firewood and water [7]. Gender-based violence is also known to be more common in conflict and post-conflict situations where male dominance is perceived to be under threat from the war and the reversed gender roles. Gender-based violence in the study area could have been increased by redundancy and readily available alcohol. In this study, we note that exposure to trauma is likely have lead to negative health behaviours like alcohol abuse which elevates gender-based violence [41].

When asked to reflect on the relationship between HRSB, well-being following and exposure to prolonged trauma, study participants recounted experiences of persistent worries and alcohol abuse. Some features indicative of depressive episodes notably; thinking a lot, which is strongly associated with clinical depression in Uganda [56,57] were noted. Others study participants were in despair, hopeless, had suicidal ideas and were pathologically fearful. The implication of all this is that untreated and unattended to mental distress associated with prolonged exposure to traumatic conflicts may not only have predisposed people to HRSB but could also has been a hindrance to the uptake of HIV preventative and treatment services.

### Methodological challenges and how they were addressed

Some methodological issues should be borne in mind when making sense of data collected using three different but related methods. The mobilization of study participants relied on opinion leaders, community members and guides from TPO-Uganda as gate-keepers. It is possible that these gate-keepers who were influential in society introduced some level of selection bias. However, such a bias was minimized if not avoided by the research team through purposeful sampling of study participants selected by the gate-keepers. The research team did a purposeful selection of study participants to remain with those that were information-rich about the study issues.

Although use of the three data collection methods was labour intensive and time-consuming, the approach enabled triangulation of data sources and improved the credibility of findings. The mixture of the methods ensured that complementary data was collected from a variety of individuals, village experts, and community members to explore issues in more depth and to build a community level perspective. Although discussions on sexuality tend to be private and closed in most African societies, this study reduced the effect of this challenge by making the discussion questions less personal and more about other people in the community.

Given vulnerability of study participants, it was highly likely to misconstrue the researchers as designated representatives of TPO-Uganda, the non-governmental organization which was implementing psychosocial programmes in the study area and which partly sponsored the study. This could explain their reception that was often cordial and anticipatory in nature. Even after self-introductions where emphasis was on the purpose of the study, most respondents still projected poverty as a central theme in their lives. Therefore, it is possible that in their responses, there could have been attempts to either please the researchers or to elicit compassion which might have affected the quality of data collected. The researchers had to assure study participants about who they were and the purpose of the research. Lastly, it is not possible to make generalizations from a study like this since the focus was on one ethnic group, the *Ateso*. Future research can do better by comparing perceptions in different war/conflict-affected ethnic and cultural groups.

### Implications of the study

The study demonstrated the direct and indirect effects of war trauma on people's abilities to make sexual behaviour choices during and after conflict. Conflict-related trauma appeared to have destabilized the socio-economic and cultural approaches to living. Morals of the people seemed to have been altered negatively during the ensuing conflict. Poverty appeared to have made certain sexual behaviours like the high turnover of sexual partners, multiple sexual partners, transactional and cross-generational sex to be tolerable. Loss of major means to livelihood was found to not only play a major role in making it difficult for people to recover from the trauma experienced but also to have changed people's socio-cultural dynamics making them vulnerable to HRSB. Efforts to restore and support people's livelihood like improvement of agriculture are likely to go a long way in helping.

Second, vulnerability to HRSB might have escalated during war and it will perhaps not dissipate rapidly following the subsiding of the conflict because of the profound societal changes that generally occurred. It may take some time for the entire society to heal and re-establish normalcy. This study adduces evidence to the effect that vulnerability to HRSB following exposure to war trauma is high in post-conflict societies. Therefore, HIV/AIDS prevention and management programs in such communities need to be comprehensive to reduce the factors which increase HRSB. There is need for aggressive psychosocial work to build fidelity between men and women though strategies like; HIV Counselling and Testing (HCT), discordant couple counselling, stress management skills, how to control or avoid alcohol abuse, domestic violence prevention and many others. The people who are HIV positive in post-conflict societies need support to start therapeutic self-help groups so that they can help one another as well as promote positive prevention. Besides, it is easier for policy makers, implementers and NGOs to assist organised people. Secondly, process of human development that is normatively based on international human rights standards and operationally directed to promoting and protecting human rights (a human rights approach) might be beneficial as postulated by some scholars [58].

The relationship between transmission of HIV/AIDS and conflict is mainly through indirect factors. War related sexual and physical trauma may not have directly increased HRSB but the consequences of war were associated with HRSB. For instance, poverty appeared to have modified morals related to sex, increased transactional sex, sexual coercion and early sexual debut. Any change agent in a post-conflict community should therefore collaborate with existing structures like religious groups, elders and opinion leaders to fight against gender-based violence, promote family values and manage alcohol abuse.

## Conclusions

The study demonstrated that conflicts disrupt the socio-cultural set up of communities and destroys sources of people's livelihood. Many people in the study area had experienced different forms of trauma associated with loss of livestock and being forced out of homes. Rehabilitation and the fight against poverty in such a community should not ignore the role played by idleness and alcohol consumption in perpetuating poverty and spread of HRSB. Post-conflict reconstruction needs to encompass programs to restructure people's morals and values through counselling as well as delivering socioeconomic services to vulnerable groups. It is imperative it to equip people with life skills to empower them so that they can make safer health choices that will promote prevention of HRSB. The findings suggests that socio-cultural dynamics which are a combination of social and cultural factors, social interactions, socio-cultural practices, power brokers, social norms and social network structures are related to the sexual health of the population. Uganda AIDS Control Program has acknowledged the role played by socio-economic deprivation in control of HIV/AIDS [59]. The added value by this study has been to expound on that role, especially in communities affected by conflict in terms of describing the underlying vulnerability factors.

More research should be done to understand the dynamics underlying HIV/AIDS services utilization, behaviour change and the interrelationship to incidence of HRSB in post conflict settings. Factors which hinder accessibility of available HIV/AIDS services to those who deserve to have them also needs to be examined to repackage effective and sustainable interventions in this and similar other settings.

## List of abbreviations

AIDS: Acquired Immune Deficiency Syndrome; CBO: Community Based Organisation; CSO: Civil Society Organisation; FGD: Focus Group Discussion; HCT: HIV Counselling and Testing; HIV: Human Immunodeficiency Virus; HRSB: High Risk Sexual Behaviour; IDP: Internally Displaced Peoples; KIs: Key Informants; LRA: Lords Resistance Army; MINI: McGill Illness Narrative Interview; PTSD: Post Traumatic Stress Disorder; STDs: Sexually Transmitted Diseases; STIs: Sexually Transmitted Infections; TB: Tuberculosis; TPO: Transcultural Psychosocial Organisation; UNAIDS: Joint United Nations Programme on HIV/AIDS; UPA: Uganda People's Army; VCT: Voluntary Counselling and Testing.

## Competing interests

The authors declare that they have no competing interests.

## Authors' contributions

All co-authors too part in the design, data collection and data analysis. WWM wrote the different drafts of the manuscript. All the other co-authors contributed by reviewing and finally approving the final version of the manuscript that was submitted.

## Authors' information

**Wilson Winstons Muhwezi**, BA (SWSA), MPhil (Health Promotion), PhD, is a senior lecturer and social worker in the Department of Psychiatry, School of Medicine, Makerere University College of Health Sciences, Kampala, Uganda.

**Eugene Kinyanda**, MBChB, M.Med (Psychiatry), PhD, is a psychiatrist and a research manager at Medical Research Council/Uganda Virus Research Institute (MRC/UVRI), Entebbe, Uganda.

**Margaret Mungherera**, MBChB, M.Med (Psychiatry), is a senior consultant psychiatrist at Mulago National Referral Hospital, and an honorary lecturer in the Department of Psychiatry, School of Medicine, Makerere University College of Health Sciences, Kampala, Uganda.

**Patrick Onyango**, BA, Msc, PGDip, is a country director, Transcultural Psychosocial Organization (TPO-Uganda).

**Emmanuel Ngabirano**, BSW, DSW, DPM, DPR, CST, MA, is a senior program officer, Transcultural Psychosocial Organization (TPO-Uganda).

**Julius Muron**, MBChB, M.Med (Psychiatry), is a psychiatrist at Butabika National Mental Referral Hospital.

**Rehema Kajungu**, BA, MA, is a program manager, Transcultural Psychosocial Organization (TPO-Uganda).

**Johnson Kagugube**, B.Stat, MA, PGDip, is Director of the District Statistics and Statistical Capacity Building, Directorate of the Uganda Bureau of Statistics (UBOS), Kampala, Uganda.
